# Therapeutic Drug Monitoring of Amikacin and Colistin in Patients with Multidrug-Resistant Gram-Negative Infections Using a Portable Plasmonic Biosensor

**DOI:** 10.1021/acs.analchem.4c06748

**Published:** 2025-06-03

**Authors:** Alejandro Astúa, Maria Carmen Estevez, Sonia Luque, Santiago Grau, Luisa Sorlí, Milagro Montero, Juan P. Horcajada, Laura M. Lechuga

**Affiliations:** a Nanobiosensors and Bioanalytical Applications Group, Catalan Institute of Nanoscience and Nanotechnology (ICN2), CSIC, CIBER-BBN, BIST, Bellaterra 08193, Spain; b Pharmacy Service, Hospital del Mar, Hospital del Mar Research Institute, Universitat Pompeu Fabra (UPF), Barcelona 08003, Spain; c Infectious Diseases Service, Hospital del Mar, Hospital del Mar Research Institute, Universitat Pompeu Fabra (UPF), Barcelona 08003, Spain; d Spanish CIBER Infectious Diseases Network, CIBERINFEC, Instituto de Salud Carlos III, Madrid 28029, Spain

## Abstract

Innovative diagnostic tools that enhance antibiotic routine monitoring can improve the management of infections caused by antibiotic-resistant bacteria. Therapeutic drug monitoring (TDM) involves measuring drug levels in the patient bloodstream to ensure optimal efficacy and safety, particularly for drugs with a narrow therapeutic index (TI), assisting in dosage control and toxicity risk management. Amikacin (AK) and colistin (CS) are crucial antibiotics for treating multidrug-resistant (MDR) bacteria but they have side effects that require a precise TDM to try to minimize them. Current analytical techniques like immunoassays, high-performance liquid chromatography (HPLC), and liquid chromatography–mass spectrometry (LC-MS) are gold standards for the antibiotic analysis, but they may require transferring the human samples to centralized facilities, delaying crucial results and turnaround time. In contrast, plasmonic biosensors offer advantages for clinical diagnostics, enabling real-time drug detection with minimal sample volume and processing, being ideal for point-of-care applications. We have implemented plasmonic biosensors to quantify and rapidly monitor blood levels of amikacin and colistin. The biosensors provide high specificity and sensitivity, with limits of detection (LOD) of 0.92 ng/mL (1.57 nM) for amikacin and 9.11 pg/mL (7.88 pM) for colistin in blood serum. Statistics analyses demonstrated a strong correlation between the biosensor evaluation and the standard analytical methods (Spearman’s correlation coefficient of 0.9171 (*p*-value < 0.001) and 0.7435 (*p*-value = 0.04) for amikacin and colistin, respectively). Our plasmonic biosensors offer in addition, simplicity, portability, and label-free evaluation, with multiplexed capabilities. The rapid turnaround of results in under 20 min, coupled with minimal sample processing, enhances the feasibility of personalized TDM, supporting tailored treatment strategies that can improve patient outcomes. This work lays the foundation for creating an integrated point-of-care biosensor platform for effectively performing TDM of antibiotics and other drugs in real-time at the patient’s bedside in clinical settings.

## Graphical abstract

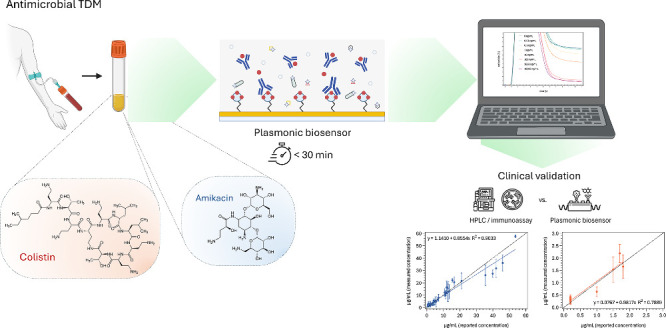

## Introduction

A significant challenge in the correct managing of bacterial infections in clinical settings is the lack of rapid accurate diagnostics. This leads to the unnecessary use of broad-spectrum first-line antibiotics until the pathogen and its antimicrobial resistance profile are identified, delaying the administration of the suitable treatment, and leading to uncontrolled and inappropriate dosage efficacy throughout the treatment cycle.^1,2^ As of 2022, antimicrobial resistance (AMR) was responsible for approximately 1.3 million deaths worldwide, with this number projected to reach 10 million by 2050, surpassing other major causes of death.^3^ The WHO has warned that multidrug-resistant (MDR) bacteria could become the next global pandemic.^4^ This crisis originates from the overuse and improper dosing of antibiotics, which has driven the emergence and spread of resistant bacterial strains.^5^ Therefore, controlling and monitoring antibiotic use is essential to mitigate the AMR problem. Part of this collective effort can be supported by dedicated therapeutic drug monitoring (TDM) that is an essential tool to select the most effective and safe dosage regimens based on the pharmacokinetics and pharmacodynamics (PK/PD) of antimicrobials.

TDM involves multiple clinical practices, including regular blood sampling, drug assays using analytical techniques, PK modeling, estimation of PK/PD targets and dose adjustment based on drug concentrations recommended by clinical pharmacists to ensure optimal drug efficacy and safety. These practices are vital for maintaining therapeutic drug levels within a target range to effectively reduce the initial bacterial inoculum, avoid toxicity and prevent the emergence of resistance. Dosage decisions depend on several factors, including patient characteristics, their renal and hepatic function, body weight, homeostasis, source of infection, and type of bacteria.^6^ A comprehensive and multidisciplinary approach is necessary to account for all these variables and tailor antibiotic therapy to individual patient needs. In addition, some antibiotics have a narrow therapeutic index (TI), meaning that the effective antimicrobial concentration is very close to the toxic concentration. In these cases, accurate therapeutic drug monitoring of the plasma concentrations is advisable.^7,8^ Despite its potential benefits, TDM is not widely implemented and is restricted to a limited number of centralized laboratories. TDM mainly relies on chromatography, mass spectrometry, and immunoassay-based techniques. Although high-throughput technologies have improved result turnaround times, sample collection and analysis remain confined to specialized facilities, making large-scale, routine TDM unfeasible in most healthcare settings.

Multiple antibiotics are targeted for therapeutic drug monitoring, with amikacin and colistin being particularly important. Amikacin (AK) is a second-generation aminoglycoside antibiotic used to treat severe bacterial infections, especially those caused by Gram-negative bacteria. It is commonly employed in ICUs, either alone or in combination with other antibiotics such as vancomycin and Meropenem.^9^ Although amikacin-resistant bacterial strains have been reported, the antibiotic remains effective due to its high efficacy. However, TDM is indicated for amikacin because of its risks of ototoxicity and nephrotoxicity, especially when the trough concentrations are high.^9–12^ Colistin (CS), also known as polymyxin E, is effective against aerobic Gram-negative microorganisms and is considered a last-resort treatment for infections caused by MDR organisms. It has a narrow therapeutic index (2–4 μg/mL) and can cause nephrotoxicity at concentrations above 2.4 μg/mL.^13^ Colistin also exhibits significant variability in pharmacokinetics among similar patient populations.^14^ Given the increasing incidence of multidrug-resistant Gram-negative bacterial infections and limited treatment options, both antibiotics have been reintroduced in the clinical practice.^15,16^ Therefore, monitoring the blood levels of amikacin and colistin is crucial.

TDM methodologies for these antibiotics are primarily conducted in blood serum or plasma and include high-performance liquid chromatography (HPLC), liquid chromatography–mass spectrometry (LC-MS), and commercial immunoassays.^17,18^ The main drawbacks of these methods are their high costs, inability to be performed at the point of care, and the extended time required (often several hours to one or more days) to deliver results and recommendations about dosing to clinicians.^19^ A point-of-care rapid method for TDM could have a significant impact on the management of severe infections. In this context, optical biosensors represent an opportunity to develop more efficient diagnostic tools that can change infection management and enhance clinical outcomes by providing integrated, multifunctional solutions for personalized medicine. These tools could offer important advantages such as reliable real-time, label-free and the potential for onsite testing with enhanced sensitivity and specificity compared to standard techniques, while also addressing limitations like long analysis turnaround times, the need for centralized laboratories, and the requirement for specialized personnel and equipment.^20^

Herein, we present a TDM platform able to provide rapid responses based on a plasmonic biosensor. Plasmonic sensors utilize evanescent waves to analyze surface phenomena, generating a signal related to minute changes in the refractive index (RI) at the sensor surface. Plasmonic biosensors have become popular in multiple fields, including environmental protection, biological studies, food safety, and clinical diagnosis.^21–23^ Our custom-designed plasmonic biosensors have previously demonstrated excellent specificity and high sensitivity when directly testing patients samples, with limits of detection (LODs) in the nM to pM range.^24–26^ We have implemented competitive immunoassays for the detection and quantification of amikacin and colistin for therapeutic drug monitoring. The integrated biosensor platform provides excellent performance when analyzing complex matrices highlighting its suitability for the direct analysis of serum from patients undergoing antimicrobial treatment.

## Experimental Section

### Clinical Samples

Serum samples from patients treated with either amikacin or colistin (in the form of its inactive pro-drug colistin methanesulfonate sodium, CMS) were collected at the Hospital del Mar (Barcelona, Spain) from patients included in the Antimicrobial TDM program of the Hospital del Mar, as it is routinely performed. All samples were collected according to the ethical standards of the institutional and/or national research committee and the 1964 Helsinki Declaration and its later amendments or comparable ethical standards. The collection included 80 serum samples: 64 samples positive for amikacin and 16 samples positive for colistin sulfate, obtained from adult patients previously treated for bacterial infections at the hospital. All blood samples were processed to measure amikacin or colistin plasma concentrations at various times after antibiotic administration as it is recommended. Colistin concentrations were determined using a validated high-performance liquid chromatography (HPLC) method as reported by Li et al.^27^ with minor modifications as described previously by our group.^28^ Amikacin concentrations were quantified by the homogeneous particle-enhanced turbidimetric immunoassay (Cobas; Roche Diagnostics GmbH, Mannheim, Deutschland). Serum samples were further stored in a local bank at −80 °C.

### Plasmonic Biosensor and Experimental Setup

Immunoassays were conducted to evaluate the biosensor performance by immobilizing a specific biorecognition layer onto the chip surface, using a homemade sensor platform. This sensor platform, previously described^29^ (see Section S1.3 in the Supporting Information for more details and Figure S1), includes all the optical, microfluidic, and electronic components necessary to evaluate the binding kinetics and affinity of molecular interactions. Specifically, the biosensor chip is clamped in a microfluidic cell which is connected to a fluidic pump that delivers a homogeneous flow of buffer over its surface. The system incorporates injection valves connected to a sample loop (350 μL) that facilitates the loading and injection of the different solutions and samples while the buffer is constantly running. Based on the Kretschmann configuration, the sensor platform achieves surface plasmon resonance conditions via a prism coupler. Interactions on the biosensor were monitored using custom readout software that tracks the increase (or decrease in the case of desorbing events) of the normalized intensity of the reflected light vs time (reflectivity% or ΔR% vs time). Representative real-time sensorgrams are shown in Figure S1.

### Biosensor Immunoassay Development

Detection assays based on competitive immunoassays were initially optimized under standard buffer conditions (PBS). Commercial mouse monoclonal antibodies targeting AK (anti-AK) and CS (anti-CS) were employed for the assay development. Antibiotic conjugates to bovine serum albumin (BSA) or ovalbumin (OVA) were prepared and attached to the transducer surface as detailed in Section S1.2 in the Supporting Information. The optimal buffer selected for each assay was kept as the running buffer throughout all the measurements. Samples (350 μL) containing a fixed concentration of antibody for the analyte of interest (anti–AK at 8 μg/mL and anti–CS at 6 μg/mL) and varying concentrations of the analyte (AK or CS) between 10^–3^ ng/mL to 10^3^–10^4^ ng/mL were prepared and incubated for 20–25 min at room temperature depending on the assay. These samples were then loaded into the sample loop system and injected onto the biosensor surface at a constant flow rate of 20 μL/min. A 20 mM NaOH solution was injected at 25 μL/min for 30–60 s as a regeneration step. Each sample injection lasted around 20 min followed by 5 min for the regeneration step. Each biosensor chip was used 20–25 times for immunoassays without significantly altering the immobilized conjugates or assay performance. Calibration curves were generated after performing triplicate measurements of the different antibiotic concentrations.

### Assessment of the Biosensor Immunoassays with Biological Samples

An accuracy test was conducted to assess the feasibility and precision of the biosensor immunoassays for detecting and quantifying AK and CS in samples across different concentrations within the therapeutic ranges of both antibiotics. After optimizing sensitivity conditions in serum, five spiked samples, prepared with commercial human serum, were blindly tested. The biosensor response signals were used to interpolate the concentration of the analyte of interest (AK or CS) in the prepared samples.

### Clinical Validation with Real Samples

Samples from patients under amikacin or colistin treatment were evaluated with the biosensor device to determine the antibiotic levels in serum. For amikacin samples, up to 200-fold dilution was applied to fall within the assay dynamic range. Similarly, for colistin samples, a dilution up to 5000-fold was considered. The resulting response (ΔR, %) for each measured sample was used to interpolate and determine the antibiotic concentration from the generated calibration curves.

## Results and Discussion

### Development and Characterization of the Biosensor-Based Immunoassays

Given the characteristics of our plasmonic biosensor, which detects changes in the refractive index due to biomolecular interactions at the sensor surface, direct immunoassays (where antibodies are immobilized on the sensor surface) are a common strategy for direct analyte capture and quantification.^30^ Amikacin and colistin, with low molecular weights, result in modest refractive index changes in direct immunoassays, leading to low detectability and reduced sensitivity. A competitive binding assay, where the analyte in the sample competes with an immobilized antigen for antibody binding, can overcome these issues. However, immobilizing low molecular weight analytes can cause steric effects, hindering the interaction of the antibody. Instead, conjugating the analyte to a carrier protein with a high molecular mass provides greater robustness, target accessibility, and reusability for the biosensor immunoassay.^31^

We synthesized AK–BSA, CS–BSA, and CS–OVA conjugates by covalently linking amikacin or colistin amino groups to BSA/OVA via EDC chemistry using carbodiimide-based cross-linking (EDC chemistry) as described in the Section S1.4. in the Supporting Information. Excess of AK and CL were used to prevent the formation of BSA or OVA aggregates and maximize the number of covalent bonds between the carrier protein and the analyte. The antibiotic:protein molar ratios were approximately 120:1 for AK–BSA, 80:1 for CS–BSA, and 50:1 for CS–OVA, respectively. The MALDI-TOF MS analysis determined between 11 and 16 antibiotic molecules per protein unit, depending on the conjugate (see Table S1 in the Supporting Information).

Custom-modified biochips were prepared with the conjugates tailored for each case to investigate their performance (see detailed procedure in the Supporting Information). Initial observations revealed significant nonspecific binding of colistin molecules on both the nonfunctionalized surface (covered only with the SAM) and the bioreceptor-coated surface (with BSA or CS-BSA), and, to a lesser extent, to the OVA conjugate (Figure S2). Cationic peptides like polymyxins have been reported to bind to plasma proteins, including albumins like human serum albumin (HSA) and BSA.^32^ Both types of conjugates have also been previously described for the immunoassays development.^33^ BSA tends to generally have stronger interactions due to its surface charge and binding sites for hydrophobic and electrostatic interactions, which are influenced by pH and ionic strength. OVA, being smaller and more globular, and despite having a net charge similar to BSA at neutral pH, possesses a more hydrophobic surface, leading to different less complex adsorption patterns than BSA. Based on the results obtained, the OVA conjugate was selected to continue the biosensor assay development.

Direct immobilization of free AK and CS yielded lower antibody signals than conjugate-coated chips (Figure S2). Conjugates improved target accessibility for antibody interaction, supporting further biosensor assay development.

Different conditions were studied to optimize the conjugate immobilization and the reagent concentrations. The optimal conditions for AK–BSA and CS–OVA conjugates immobilization were achieved at pH 7.4 with a concentration of 200 μg/mL (Figure S3), which resulted in higher immobilization yields. The biosensor response to increasing antibody concentrations was evaluated until a saturation pattern was observed. Fixed antibody concentrations of 8 μg/mL for the AK biosensor and 6 μg/mL for the CS biosensor were selected (Figure S4) and used to develop the competitive assay. These concentrations were chosen according to the signal (high enough to facilitate optimal dynamic ranges, covering a broad range of concentrations), and below the curve saturation point to facilitate a more efficient competition.

For competitive immunoassays (Figure 1A), the response signal is inversely proportional to the concentration of free analyte incubated with the antibody in the sample. Sensorgrams showing response signals at different free analyte concentrations for both biosensors are presented in Figure 1B,C.

**Figure 1:**
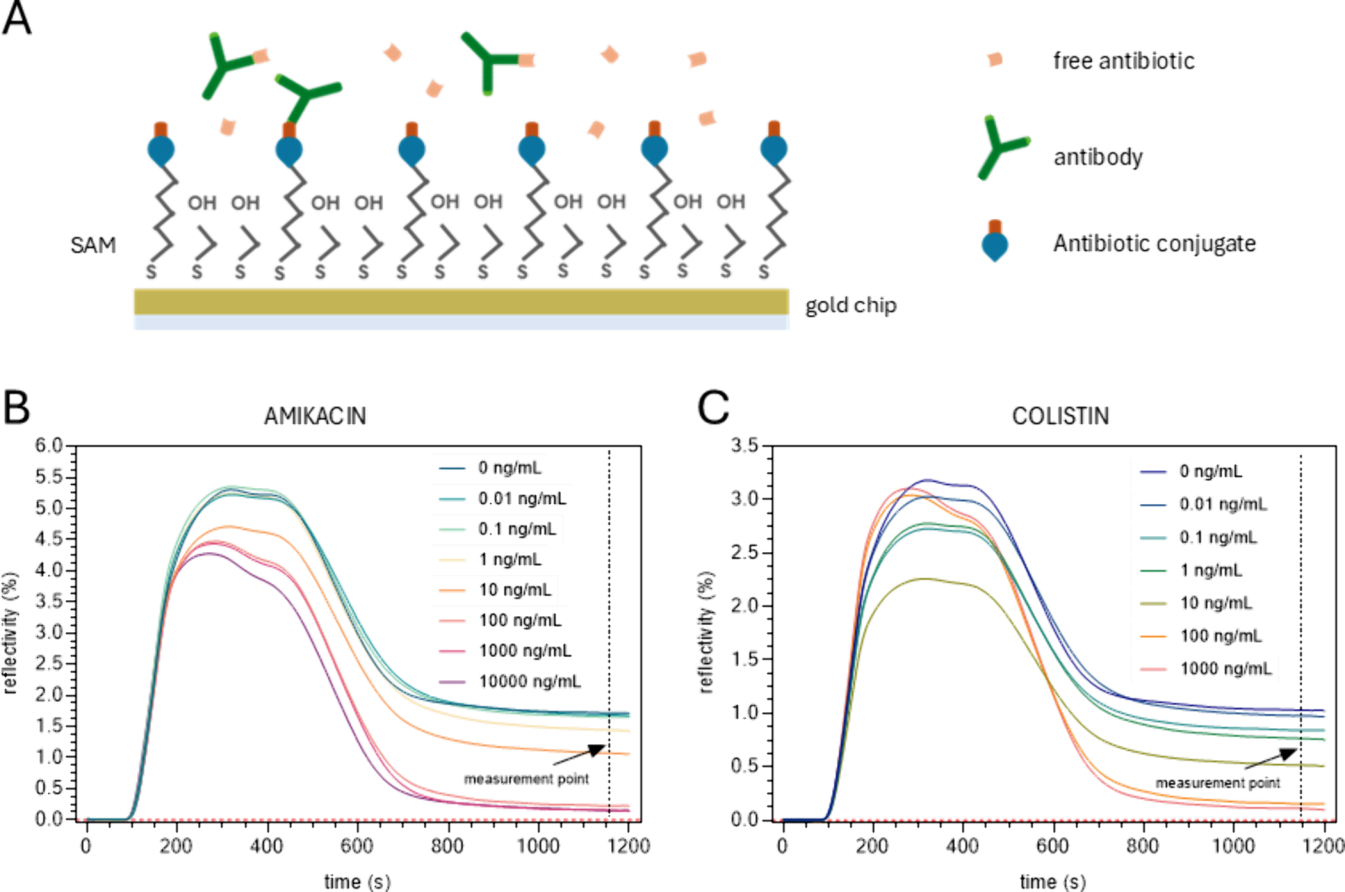
(A) Scheme of a competitive immunoassay for AK and CS determination. Real-time biosensor response for competitive immunoassays detecting varying concentrations of (B) amikacin and (C) colistin. [anti–AK] = 8 μg/mL and [anti–CS] = 6 μg/mL. The red dotted line indicates the zero signal position. The time at which the signal was recorded is also indicated by the dashed black line.

Standard calibration curves for AK and CS biosensors were generated. According to the assay analytical parameters (Table S2 and Figure S5) the LOD for the AK biosensor was 0.12 ± 0.07 ng/mL and a working range between 0.55 ± 0.27 and 14.96 ± 7.60 ng/mL. This biosensor method is highly effective for detecting AK, with sensitivity levels well below the clinical therapeutic range of 5–35 μg/mL [9]. The CS biosensor achieved a LOD of 8.04 ± 6.24 pg/mL, in the pM range (6.85 ± 5.31 pM), and a working range of 19.87 ± 9.34 – 2045.63 ± 208.94 pg/mL, demonstrating exceptional sensitivity. We have obtained a high sensitivity compared to the reported values for standard methods like LC-MS and HPLC, or immunoassays, which usually reach the nM−μM range.^34–38^ The slope of the calibration curve is lower (0.56) than the one for the AK assay (i.e., slope = 0.90), indicating that the signal change is less responsive to analyte concentration changes. This lower slope value affects the sensitivity, slightly reducing it, which may affect especially near the detection limits. Nevertheless, the biosensor assay displays a wide linear range of 2 orders of magnitude. Overall, the biosensor assay features ensure a suitable performance for detecting colistin, considering that the therapeutic range for TDM is usually between 2–4 μg/mL,^13^ which is several orders above the assay linear range, and dilutions will be necessary to measure patient samples.

### Effect of Serum on the Assay Performance and Preliminary Validation of the Biosensor

Undiluted pooled human serum was evaluated through the direct injection of a serum sample over biofunctionalized chips with the corresponding conjugates (i.e., sample with no antibody nor analyte present) resulting in high signals (reflectivity %) of 4.17 ± 1.21 for the AK biosensor and 16.71 ± 0.65 for the CS biosensor (data not shown). These results are mainly due to the matrix effect, where nontarget components like proteins and other biomolecules can adsorb onto the sensor surface (i.e., resulting in net positive changes in reflectivity), potentially masking or falsely enhancing the detection of the target analyte and affecting the biosensor capabilities for analyzing real samples. Given the high sensitivity obtained from the calibration curves in standard conditions compared with the clinical requirements, sample dilution is recommended to minimize matrix effects without compromising the biosensor performance. Dilutions up to 10,000 times for amikacin samples and 100,000 times for colistin samples should be achievable to fall in the clinical range of serum levels. However, balancing dilution with reduced nonspecific binding is crucial to maintain sample integrity. We measured the response to different serum dilutions for both biosensors. In both cases, nonspecific binding decreased as higher dilution factors were applied (see Figure S6 in SI). For the amikacin biosensor at a 1:1 and 1/10 dilution, matrix components resulted in still high nonspecific binding, thus, significantly masking the response from the specific interaction of the antibody over the bioreceptor layer. At a 1/100 dilution, the nonspecific adsorptions were minimized while the antibody interaction remained high (similar to the one obtained in standard buffer condition with a ΔR% of around 1.0). For the colistin biosensor, the undesired nonspecific adsorption was significantly higher even for 1/100 dilutions, with a significant decrease observed only at a 1/1000 serum dilution. Nevertheless, nonspecific interactions were not completely removed, thus masking the interaction of the antibody (i.e., a higher signal of serum at 1/1000 without antibody than serum 1/1000 containing the antibody). These results can be attributed to the amphiphilic nature of colistin sulfate and its precursor, CMS, which are known to form aggregates with albumins in serum.^16,33^ This aggregation promotes increased adsorption of albumin and other serum proteins on the colistin-modified biosensor surface, potentially impacting overall performance. Consequently, a 1/1000 dilution proved to be an effective initial approach to address these issues. However, the antibody response appeared partially masked at higher dilutions, indicating the need to implement and combine additional strategies.

To fully minimize the serum effect and maintain efficient antibody recognition, we tested (i) the incorporation of a blocking step and (ii) the change of the buffer composition. In the AK biosensor, the use of common blocking agents employed in immunoassays and optical label-free biosensors did not prevent the nonspecific binding nor improve the binding of the antibody (see Table S3). Blocking with 5% BSA was the only method that effectively reduced nonspecific interactions from serum. However, it also interfered with antibody binding, possibly by hindering access to the amikacin units on the sensor surface. For the CS biosensor, none of the blocking agents reduced nonspecific adsorption of 1/1000-diluted serum; in fact, BSA and OVA blocking increased it, likely due to interactions between colistin on the immobilized conjugate an additional layer of albumin molecules. Moreover, in the CS biosensor, the recognition of the antibody was significantly affected in all cases, observing a reduced binding signal (see Table S4).

Based on these results, no further blocking was applied. Instead, the buffer composition was modified as a second approach to investigate the impact of different additives. The main results for these tests are shown in Figure 2 and Figure S7. For the amikacin biosensor, the optimal results were obtained by adding Tween-20 at 0.10% (v/v) and increasing salt concentration to a final concentration of NaCl 500 mM. This approach minimized nonspecific adsorptions while enhancing antibody sensitivity to amikacin. Increasing NaCl concentration likely screened electrostatic interactions, reducing nonspecific binding, while Tween-20, a nonionic surfactant, reduced hydrophobic interactions and acted as a barrier to nonspecific adsorptions. These additives synergistically improved the biosensor specificity and sensitivity by ensuring that only specific binding events contributed to the signal.

**Figure 2:**
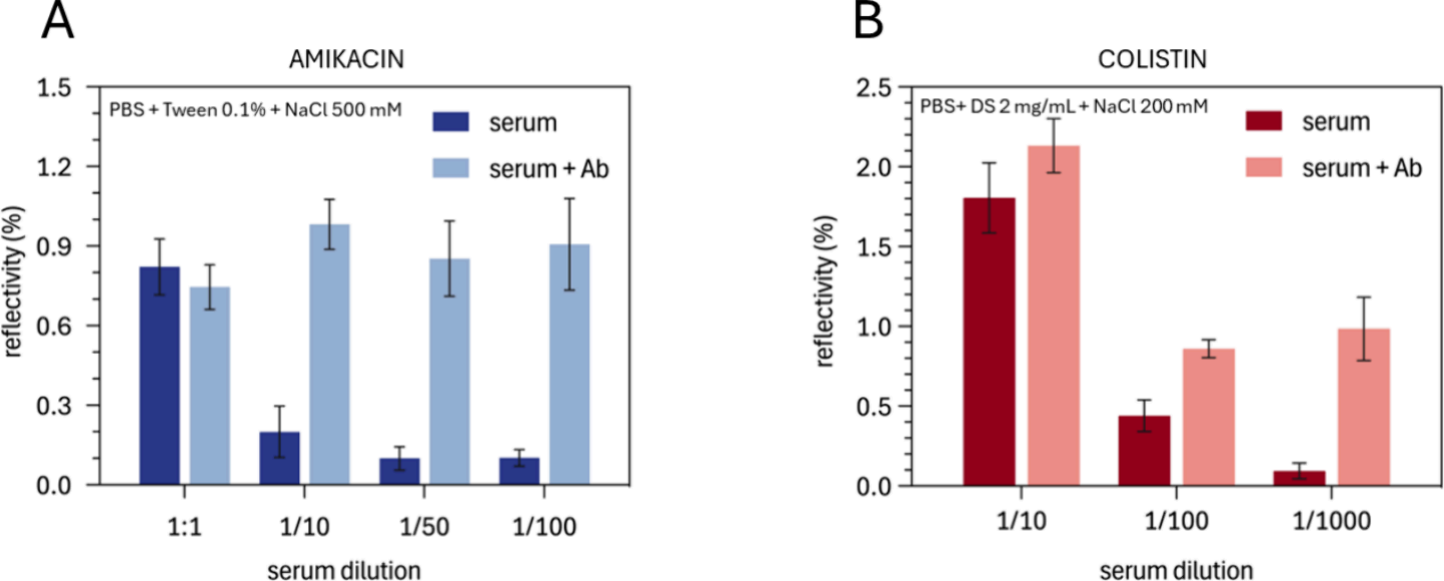
Biosensor response to different serum dilutions without and with each corresponding specific antibody for (A) amikacin and (B) colistin biosensors. [anti–AK] = 8 μg/mL; [anti–CS] = 6 μg/mL. The final buffer composition to dilute the serum is shown in each case. The data correspond to the average ± SD of duplicates.

For the CS biosensor, optimal sensitivity and minimal nonspecific binding were achieved by combining PBS buffer with dextran sulfate (DS) at 2 mg/mL and increasing NaCl concentration to 200 mM. The higher salt concentration helped reduce nonspecific interactions by enhancing ionic strength, while dextran sulfate, a polyanionic polysaccharide, likely further reduced nonspecific binding through electrostatic repulsion. This prevented unwanted adsorption of colistin molecules and other matrix components to the carrier protein (OVA) immobilized on the sensor surface. Additionally, DS may have interacted with the cationic part of colistin via electrostatic attraction, forming complexes that stabilized colistin and enhanced assay specificity by minimizing nonspecific interactions.

According to this, calibration curves in the minimum viable serum dilution for each antibiotic (1/10 and 1/1000 for AK and CS, respectively) were obtained employing the optimal buffer composition: for the AK biosensor, samples were diluted 1/10 in PBST 0.10%, pH 7.4, and 500 mM NaCl. For the CS biosensor, PBS pH 7.4 with 200 mM NaCl and DS 2 mg/mL was used to dilute a minimum of 1/1000. Calibration curves are shown in Figure 3, with assay parameters in Table 1. Specificity was evaluated by running samples containing vancomycin or Meropenem as negative controls, considering these antibiotics can be coadministered with either amikacin or colistin.

**Figure 3:**
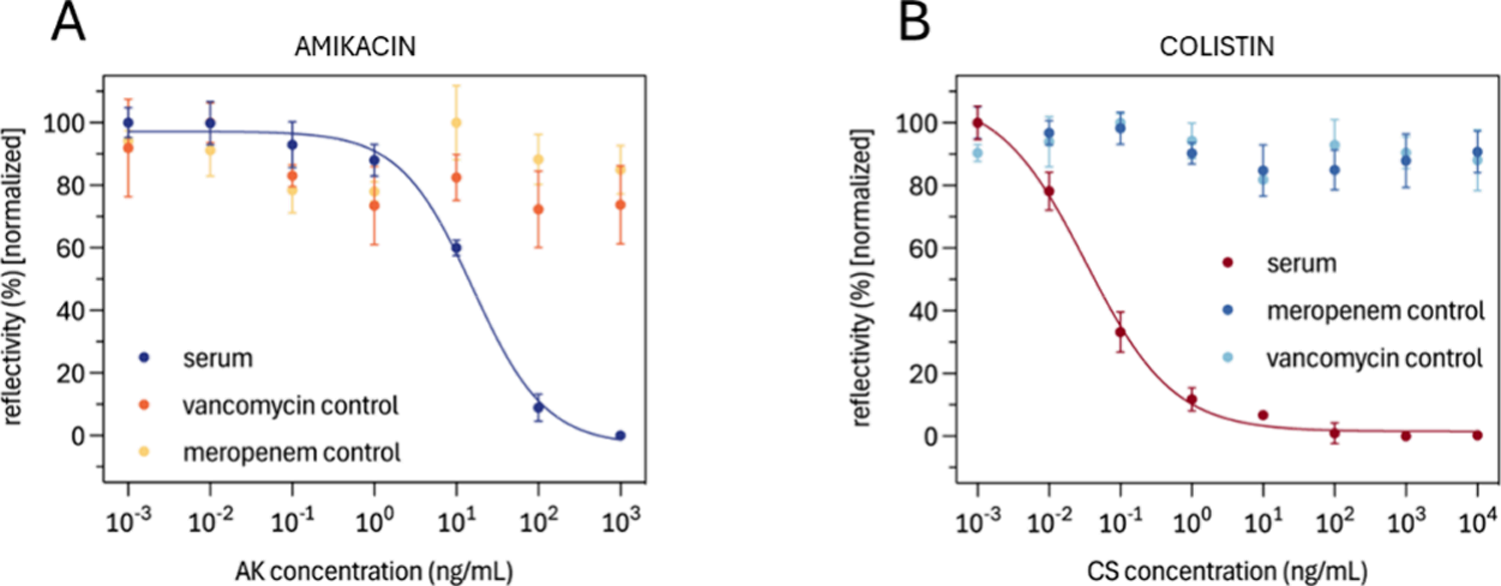
Normalized calibration curves in diluted serum obtained for (A) amikacin and (B) colistin biosensors. Vancomycin and Meropenem negative controls are also shown in both curves. The data correspond to the average ± SD of triplicate measurements.

**Table 1:** Plasmonic-Based Immunoassay Parameters for Amikacin and Colistin in Diluted Serum^c^

	**amikacin biosensor**	**colistin biosensor**
	serum^a^	serum^b^
LOD (IC_90_)	0.92 ± 0.47 ng/mL	3.77 ± 0.36 pg/mL
	(1.57 ± 0.80 nM)	(3.21 ± 0.31 pM)
IC_50_	11.3 ± 1.87 ng/mL	43.5 ± 2.05 pg/mL
	(19.36 ± 3.19 nM)	(37.0 ± 1.75 pM)
working range (IC_80_– IC_20_)	2.42 ± 0.48 – 37.5 ± 4.12 ng/mL	8.11 ± 1.10 – 300.7 ± 29.8 pg/mL
	(4.13 ± 0.82 – 64.05 ± 7.04 nM)	(6.90 ± 0.94 – 256.0 ± 25.3 pM)
slope	–1.01 ± 0.15	–0.71 ± 0.08

a 1/10 serum dilution in PBST 0.10%, pH 7.4, and 500 mM NaCl.

b 1/1000 serum dilution in PBS pH 7.4 and 200 mM NaCl + DS 2 mg/mL.

c Data obtained from triplicate measurements.

The AK biosensor showed a slight decrease in detectability in serum, with a reduced working range compared to buffer conditions. However, the slope of the curve in serum was improved relative to that in buffer, probably because of the additives used. The parameters obtained in serum are suitable for interpolating amikacin concentrations in real samples, as they fall within the therapeutic range needed for TDM. Similarly, the CS biosensor in serum shows a higher IC_50_ and a narrower working range. Despite a slight reduction in sensitivity and measurement range, the analytical performance of the biosensor assay remains sufficient to detect the antibiotic well within the limits for effective monitoring.

Table S5 shows intra- and interassay variability. The AK biosensor displays slightly higher LOD variability than the CS biosensor, with intra-assay %CVs of 12.1% vs 8.7%, and interassay %CVs of 17.0% vs 15.9%, respectively. While both biosensors demonstrate acceptable repeatability, the slightly higher LOD variability for amikacin suggests reduced consistency across different runs. Nonetheless, the precision levels for both biosensors remain below 20%, the commonly accepted threshold for immunoassay validation, making them suitable for reliable TDM applications.

Finally, an accuracy study was conducted with spiked samples prepared for both types of biosensors by adding a defined concentration of analyte (either AK or CS) in undiluted serum. Results are shown in Table 2. For the amikacin samples, the plasmonic biosensor delivered a highly sensitive and accurate detection assay, reliably detecting amikacin in the range of 5–30 μg/mL for TDM purposes. For colistin, the biosensor exhibited limited capacity to detect the antibiotic at low concentrations but demonstrated better accuracy at higher levels. It remained effective within the TDM range of 1–4 μg/mL. Given the significant interest in measuring samples around 2.4 μg/mL or above, which is the reported concentration associated with nephrotoxic effects in patients,^13^ the biosensor could be effectively used for this purpose.

**Table 2:** Accuracy Study of the Amikacin and Colistin Biosensors with Serum Spiked Samples

**sample** ^a^	**real concentration (ng/mL)**	**plasmonic biosensor (ng/mL)** ^b^	**accuracy (%)**
AK01	30.0	28.6 ± 6.7	95
AK02	2.0	1.8 ± 1.0	92
AK03	15.0	11.2 ± 2.5	74
AK04	20,000.0	20,074.0 ± 408.7	101
AK05	50,000.0	46,964.0 ± 5,811.0	94
CS01	8.0	2.7 ± 1.4	34
CS02	40.0	35.8 ± 7.9	90
CS03	1.5	0.4 ± 0.5	27
CS04	3,000.0	2,780.0 ± 469.5	93
CS05	6,000.0	5,210.0 ± 785.7	87

a AK01–AK05: amikacin blind samples; CS01–CS05: colistin blind samples.

b Samples were measured by duplicate.

### Clinical Validation of the Biosensor with Patient Samples

A total of 80 clinical samples were analyzed for the validation of both biosensors, including 64 samples positive for amikacin and 16 samples positive for colistin, collected from patients treated for infections at Hospital del Mar (Barcelona, Spain). Serum samples were collected at peak concentration (approximately 15–30 min after the antibiotic infusion was completed) or at trough concentration (just before the next dose was administered). Samples were analyzed at the Pharmacy Service of Hospital del Mar using the standardized methods for each antibiotic. Samples were diluted to fall within the working range of the assays. The results are summarized in Figure 4 and Tables S6 and S7.

**Figure 4:**
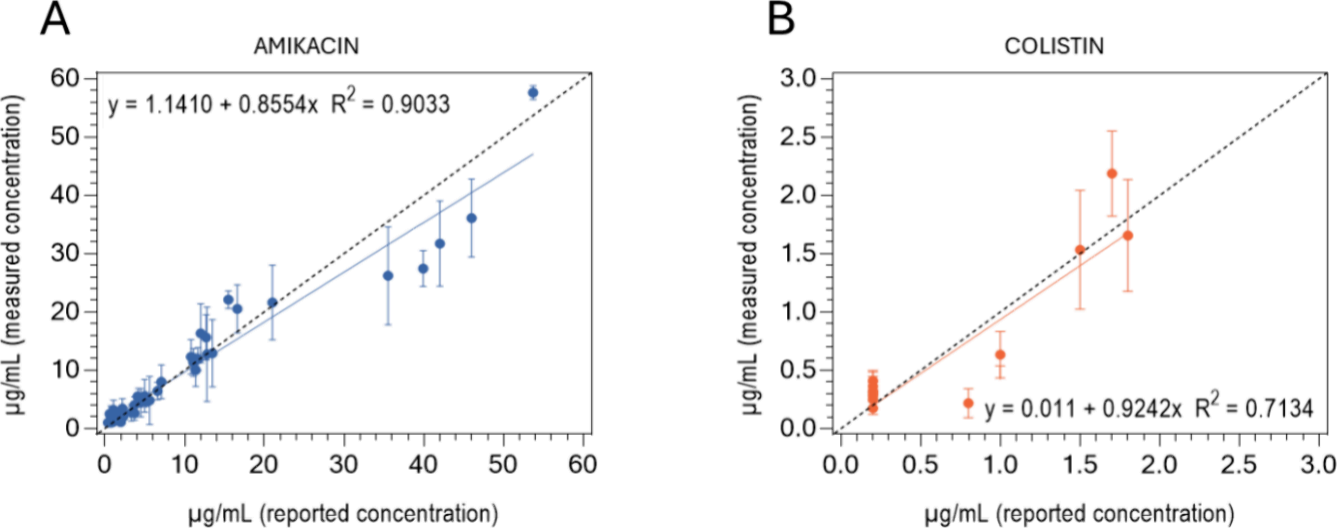
Accuracy studies of the (A) amikacin and (B) colistin biosensors. Linear regression analysis comparing antibiotic concentrations obtained using the biosensor (measured concentration) with those determined by the standard methods (reported concentration). Dashed line represents a perfect correlation between both techniques (slope = 1). The data correspond to the average ± SD of duplicates.

In the case of the AK biosensor, the correlation between both methods is strong (R^2^ = 0.9033), indicating a reliable relationship. The slope of 0.85 suggests a slight underestimation compared to the standard method, but the overall trend remains consistent. Greater discrepancies were observed in the quantification results at higher amikacin concentrations, possibly due to saturation effects. For the CS biosensor, the fit between both methods showed a moderate correlation (R^2^ = 0.71) with a slope of 0.924, indicating a general alignment with the standard method. Similarly, larger variances were observed between measurements of the same sample at higher CS concentrations. This could be due to varying interactions between CS in real samples and the buffer or the bioreceptor layer.

Regarding the sensitivity comparison and accuracy between the detection assays, the limits of quantification (LOQ) reported for amikacin and colistin using the turbidimetric immunoassay and HPLC method are 0.8 μg/mL and 0.2 μg/mL, respectively.^39^ For amikacin samples near the LOQ, the biosensor showed an overestimation in quantification. In contrast, the CS biosensor showed relatively good accuracy for concentrations near the LOQ, indicating the biosensor capability to quantify both below and within the therapeutic range required for TDM.

To evaluate the consistency of our plasmonic biosensors, we conducted a nonparametric correlation analysis using Spearman’s rank correlation coefficient. The results are presented in Figure S8. The coefficients obtained were 0.9171 for AK (*p*-value of <0.001) and 0.7435 for CS (*p*-value of 0.04), respectively. These values indicate an excellent agreement between the quantification results obtained with the standard method and the concentration values determined with the developed biosensor, especially in the case of amikacin. However, due to the high variance in accuracy and standard deviation observed in individual samples, we also conducted a Bland-Altman analysis to further compare our proposed biosensor method for quantifying antibiotics. The results are shown in Figure 5 and Table S8.

**Figure 5:**
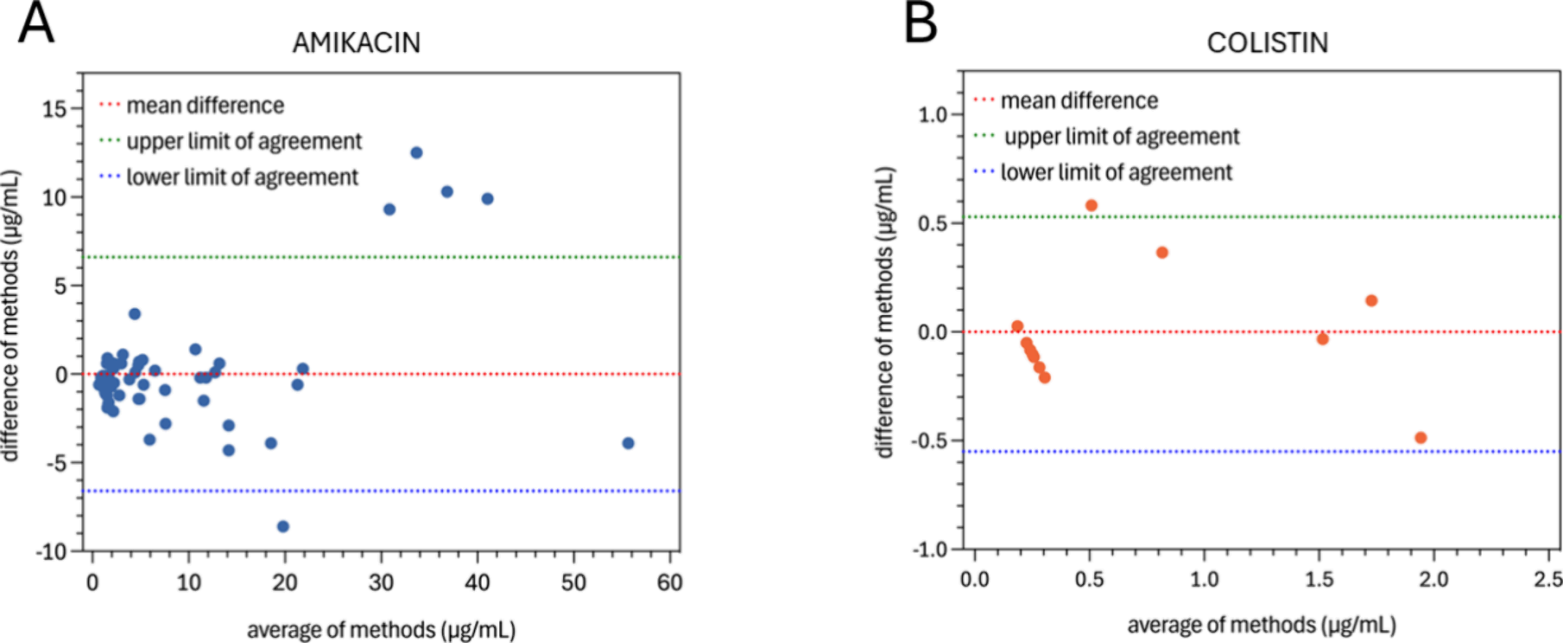
Distribution of sample measurements according to the Bland-Altman test for (A) amikacin and (B) colistin serum samples.

The Bland-Altman analysis is a statistical method used to assess the agreement between two measurement techniques. It not only quantifies the strength of the relationship between the measurements, like Spearman’s coefficient, but also analyzes the systematic and random differences between the methods, providing insights into possible bias, variability, and outliers across different measurement ranges.^40^ This is relevant for our biosensors as regression analysis showed differences in accuracy and standard deviation across concentration ranges for both biosensors.

For the amikacin biosensor, the t-statistic was −0.3322 with a *p*-value of 0.7409, indicating no statistically significant difference between the biosensor and the HPLC method. For the colistin biosensor, the t-statistic was −1.0048 with a *p*-value of 0.3387, also indicating no statistically significant difference between the two methods. However, the results reveal that the new biosensor consistently overestimates concentrations (systematic bias) and shows increased variability at lower concentrations, especially in samples where the reported concentration was less than 0.8 μg/mL for AK or 0.2 μg/mL for CS.

Based on these analyses, our biosensors can better quantify AK concentrations at higher levels relevant for TDM, though with increased variability. Conversely, the CS biosensor showed better accuracy at higher concentrations. In this sense, it is known that colistin easily adheres to glass and polymer surfaces, including polypropylene and polystyrene.^41^ The amphiphilic structure of colistin, comprising a hydrophobic cyclic heptapeptide and hydrophilic amino groups, may have partially influenced its interactions and the results obtained from the biosensor immunoassay. Here, it is worth mentioning that only 14 samples were analyzed for colistin, and considering the complexity of its handling, further research with a higher number of samples could be beneficial to enhance the biosensor measurement capabilities.

Overall, the results obtained demonstrate the performance provided by both plasmonic biosensors, with the quantification of antibiotics in a 20 min assay time, with diagnostic reliability equivalent to standard methods, being particularly beneficial in the case of the HPLC measurements, which rely on complex sample preparation before analysis. Our findings highlight the potential and benefits of rapid, label-free, and simple optical biosensor technologies in clinical settings for routine drug measurement directly in patient samples. The developed plasmonic biosensors for amikacin and colistin offer a promising alternative for TDM, potentially enhancing the efficiency and accessibility of antibiotic monitoring in clinical practice.

## Conclusions

We have developed highly sensitive, rapid, and label-free plasmonic biosensors for the therapeutic monitoring of amikacin and colistin. These biosensors require only a small sample volume of a few microliters, making them suitable for clinical settings. The feasibility of measuring serum, which only requires simple dilution, simplifies the pretreatment and shortens the overall sample processing and analysis time, enabling semicontinuous monitoring. Although the current measurements were performed in serum, ideally, direct blood analysis would be preferred to bring the biosensor closer to a point-of-care (POC) format. The system, however, can be adapted to integrate filtration methods that eliminate the need for centrifugation, making it more practical for decentralized applications. The detection strategy uses a competitive immunoassay with conjugates of AK or CS immobilized on the sensor chip surface. The immobilization process and other assay parameters were optimized, resulting in promising sensitivity and specificity in both buffer conditions and biological samples. The achieved LODs were in the ng/mL range for the AK biosensor and the pg/mL range for the CS biosensor. While these LODs are notably low, the need for significant sample dilution places most measured concentrations at the higher end of the assay’s range. Nevertheless, the method remains effective for detecting antibiotics within clinically relevant concentrations. Both biosensors were validated using real serum samples from patients undergoing infection treatment. We observed a positive correlation and agreement between our method and standard techniques used for antibiotic quantification. However, the accuracy and other descriptive statistics indicated variability in quantifying low and high antibiotic concentrations, which is crucial when considering expected antibiotic levels or monitoring different stages of the dosing cycle. Despite these differences, our results confirm the potential of plasmonic biosensors as a promising tool for TDM enabling high-frequency sampling and real-time information on antimicrobial levels in patients. This rapid monitoring has the potential to optimize dosing strategies and improve personalized treatment, reducing risk of toxicity or therapeutic failure.

## Supplementary Material



## Notes

Detailed reagents, experimental protocols for conjugates preparation, surface biofunctionalization, and data analysis; impact of different assay conditions on analytical performance; effect of serum on assay performance; complementary data on real sample analysis and clinical validation (PDF)
