# Supplementary material for: Therapeutic Drug Monitoring of Amikacin and Colistin in Patients with Multidrug-Resistant Gram-Negative Infections Using a Portable Plasmonic Biosensor
Source: Anal Chem. 2025 Jun 3;97(23):12051–9. doi: 10.1021/acs.analchem.4c06748 (PMC12177867; doi:10.1021/acs.analchem.4c06748)
Supplement: Supplementary file 1 [file ac4c06748_si_001.pdf]

SUPPORTING INFORMATION

Therapeutic Drug Monitoring of Amikacin and Colistin in Patients with Multidrug-Resistant Gram-Negative Infections Using a Portable Plasmonic Biosensor

Alejandro Astúa [a], *Maria Carmen Estevez* [a*], *Sonia Luque* [b], *Santiago Grau* [b], *Luisa Sorlí* [c], *Milagro Montero* [c], *Juan P. Horcajada* [c], *Laura M. Lechuga* [a]

[a] *Nanobiosensors and Bioanalytical Applications Group, Catalan Institute of Nanoscience and Nanotechnology (ICN2), CSIC, CIBER-BBN, BIST, 08193 Bellaterra, Spain.*

[b] *Pharmacy Service, Hospital del Mar, Hospital del Mar Research Institute, Universitat Pompeu Fabra (UPF), Barcelona, Spain. Spanish CIBER Infectious Diseases Network. CIBERINFEC. Instituto de Salud Carlos III, Madrid, Spain*

[c] *Infectious Diseases Service, Hospital del Mar, Hospital del Mar Research Institute, Universitat Pompeu Fabra (UPF), Barcelona, Spain. Spanish CIBER Infectious Diseases Network. CIBERINFEC. Instituto de Salud Carlos III, Madrid, Spain*

*Corresponding author: Maria Carmen Estévez, Catalan Institute of Nanoscience and Nanotechnology (ICN2), 08193 Bellaterra, Spain. Email: mcarmen.estevez@icn2.cat

TABLE OF CONTENTS

Section 1. Materials and Methods **S-3**

S1.1. Chemical and immunoreagents **S-3**

S1.2. Synthesis of antibiotics conjugates **S-3**

S1.3. Plasmonic biosensor device and signal interpretation **S-4**

S1.4. Surface biofunctionalization **S-4**

S1.5. Data analysis **S-5**

Section 2. Figures **S-6**

Figure S1: Scheme of the plasmonic device and biosensing principle **S-6**

Figure S2. Nonspecific adsorption of antibiotics over functionalized sensor chips **S-7**

Figure S3: Effect of pH and conjugate concentration on the biofunctionalization **S-8**

Figure S4. Non-competitive saturation curves for AK and CL assays **S-9**

Figure S5. Standard calibration curves for AK and CL in buffer conditions **S-9**

Figure S6: Effect of serum in amikacin and colistin assays **S-10**

Figure S7: Effect of additives on nonspecific adsorptions in diluted serum **S-11**

Figure S8: Data correlation of real samples **S-12**

Section 3. Tables **S-13**

Table S1: Characterization of antibiotic conjugates **S-13**

Table S2: Biosensor immunoassay for amikacin and colistin in buffer **S-14**

Table S3: Blocking agents study for the amikacin biosensor in serum 1/10 **S-15**

Table S4. Blocking agents study for the colistin biosensor in serum 1/1000 **S-16**

Table S5: Intra-assay and inter-assay variability of assays in diluted serum **S-17**

Table S6: Quantification of amikacin concentrations in human serum samples **S-18**

Table S7: Quantification of colistin concentrations in human serum samples **S-20**

Table S8: Bland-Altman analysis and performance metrics **S-21**

S1. Materials and methods

S1.1. Chemicals and immunoreagents

Reagents for self-assembled monolayers (SAMs), including 11-mercaptoundecanol (MUOH) and 16-mercaptohexadecanoic acid (MHDA), were obtained from Sigma-Aldrich (Madrid, Spain). Additional reagents for buffer preparation, surface chip biofunctionalization, and immunoassays were also purchased from Sigma-Aldrich. These included PBS tablets (10 mM phosphate buffer, 2.7 mM potassium chloride, and 137 mM sodium chloride, pH 7.4), MES, 1-ethyl-3(3-dimethylaminopropyl)-carbodiimide hydrochloride (EDC), sodium N-hydroxysulfosuccinimide (NHS), ethanolamine hydrochloride (>99%), sodium chloride (≥99%), sodium hydroxide (NaOH, anhydrous, >98%), dextran sulfate sodium salt (DS, MW ~40,000), bovine serum albumin (BSA), albumin from chicken egg white (OVA), Tween-20, amikacin (AK), colistin sulfate (CS), vancomycin hydrochloride (VC), and meropenem (MP). Antibodies specific for amikacin (mouse monoclonal IgG anti-amikacin, anti–AK) and colistin sulfate (mouse monoclonal IgG anti-polymyxin E, anti–CS) were purchased from Fitzgerald Industries (Acton, MA, US) and Creative Diagnostics (Shirley, NY, US), respectively. Pooled human AB serum was acquired from Innovative Research (Novi, MI, US) and stored at -20 °C.

S1.2. Synthesis of antibiotics conjugates

The amikacin conjugate (AK–BSA) was prepared by coupling the amino groups of AK to the carboxyl groups (Glu and Asp amino acid residues present in BSA). Briefly, a solution (final volume of 1 mL) of AK (11.0 mg/mL in 50 mM MES buffer, pH 5.0) and the carrier protein BSA (10 mg/mL in Milli-Q water) was incubated for 3 hours at 25 °C with 10 mg/mL of EDC (in 50 mM MES buffer, pH 5.0). The BSA conjugate was purified from excess reagents by size filtration using a 30 kDa centrifugal filter (Amicon® Ultra 30K, Merck KGaA, Darmstadt, Germany) and PBS, according to the manufacturer's instructions. The solution was mixed with 250 µL of buffer in the filter and centrifuged at 12,000 rpm at 25 °C for 120 seconds, after which the residues from the collection tube were discarded. This process was repeated three times, yielding the final conjugate solution. The conjugate concentration was determined by UV spectrophotometry (SpectraMax iD3, Molecular Devices, San Jose, CA, US). Colistin conjugates (CS–BSA and CS–OVA) were prepared similarly, using either BSA or OVA as the carrier protein. The procedure was the same as described above (with a final solution volume of 1 mL), using 15.5 mg/mL of CS, 10 mg/mL of BSA or OVA, and 10 mg/mL of EDC, all diluted in 50 mM MES buffer with 500 mM NaCl, pH 6.0. The final concentration of BSA–OVA and CS–OVA conjugates was also determined by UV spectrophotometry. MALDI-TOF

mass spectrometry analysis was conducted for all the conjugates to determine the number of amikacin and colistin molecules bound to the carrier proteins.

S1.3. Plasmonic biosensor device and signal interpretation

The compact, in-house plasmonic biosensor is based on the Kretschmann configuration for real-time detection of biomolecular interactions. The device measures reflectance at a fixed incident angle and features dual measurement channels, enabling simultaneous sample analysis and reference measurements. A 670 nm laser diode and a custom beam splitter direct light through an optical coupling prism onto a gold-coated sensor chip. The system operates in two modes: angular scanning, which determines the optimal resonance angle, and fixed-angle measurement, which monitors refractive index changes in real-time. The latter, offering faster interpretation and kinetic analysis, is the primary method employed here for biosensing measurements. A schematic image of the optical platform and the signal interpretation is shown in the Figure S1.

S1.4. Surface biofunctionalization

The gold chips used for the plasmonic biosensor consisted of a glass surface coated with 1.5 nm of titanium and 48 nm of gold, with dimensions of 10×10×0.3 mm, provided by Ssens (Enschede, Netherlands). The sensor chips were prepared for immobilization by cleaning them with acetone, ethanol, and Milli-Q water in a sonicator for 1 minute in each solvent. This was followed by a 30 min surface cleansing with a UV-ozone cleaner (Bioforce Nanosciences, Ames, IA, US) to remove any organic contamination. After cleaning, the sensor chips were dried with N_2. A covalent strategy was used for surface biofunctionalization. First, an alkanethiol self-assembled monolayer (SAM) was formed by immersing the gold chip overnight with a mixed solution of MHDA/MUOH in absolute ethanol, with a molar ratio of 1:5 and a total concentration of 1 mM. The sensor chip was rinsed with ethanol, dried with N_2, and mounted onto the plasmonic platform to monitor bioreceptor immobilization in real-time. Milli-Q water was used as a continuous flow solution. Either the AK–BSA or CS–OVA conjugate was covalently coupled to the carboxylic groups of the SAM through activation with a solution of 0.2 M EDC/ 0.05 M NHS in 100 mM MES (pH 5.5), run at 15 µL/min. Subsequently, a solution in PBS buffer containing either the AK–BSA conjugate (200 µg/mL) or CS–OVA conjugate (200 µg/mL) was injected at 10 µL/min. Finally, an ethanolamine solution (1 M, pH 8.1) was passed through to deactivate remaining activated carboxylic groups at 15 µL/min for 120 seconds. An indirect competitive immunoassay was used for the detection

of AK and CS. After the conjugate immobilization, Milli-Q water was replaced with PBS as the running buffer in the plasmonic biosensor.

S1.5. Data analysis

Real-time sensorgrams were processed by extracting the change in reflectivity (ΔR, %) after signal stabilization, once the entire sample passed through the flow cell. Data processing and statistical analyses were conducted using Origin 2019b software (OriginLab Corp., MA, US), Graphpad Prism 10 (Graphpad Software Inc., CA, USA), and JupyterLab 4 (Jupyter Project, CA, US). The biosensor response was represented by expressing the change in reflectivity (ΔR, %) of each standard point. Calibration curves for both amikacin and colistin were obtained as the mean and standard deviation (mean ± SD) of the signal versus the logarithm of the antibiotic concentration. Data were fitted to a four-parameter logistic regression (4PL) equation according to the following formula:

$$y = D + \frac{A - D}{1 + \left(\frac{x}{C}\right)^{B}} \qquad \text{Equation S1}$$

In this equation, *y* corresponds to the biosensor response (ΔR, %), *x* is the antibiotic concentration (in ng/mL), *A* represents the asymptotic maximum value corresponding to the signal in the absence of the antibiotic, *B* is the slope of the curve at the inflection point, *C* represents the inflection point, which is equivalent to the half-maximal inhibitory concentration (IC_{50}), and *D* is the asymptotic minimum value, corresponding to the background signal. The limit of detection (LOD) was calculated as the antibiotic concentration corresponding to 90% of the signal (IC_{90}). The working range was set as the interval between 20% and 80% of the normalized signal (IC_{20}–IC_{80}). The correlation between concentration obtained with the plasmonic biosensor and the reported concentrations using the standard analytical methods was analyzed using a Spearman test. Further statistical analyses, including an agreement analysis and a Bland-Altman test, were conducted to evaluate the potential of the designed biosensors to evaluate real samples containing amikacin or colistin in a clinical setting.

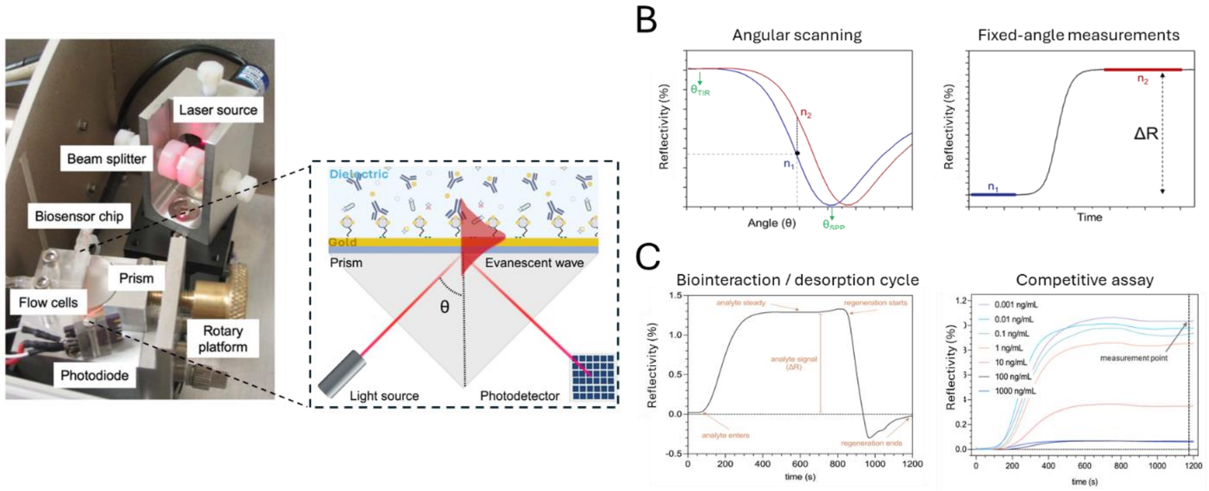

Figure S1. (A) Optical subsystem of the plasmonic biosensor, which includes the laser, beam splitter, prism as coupling element, adjustment system, photodetector, and rotatory platform for angular scanning. A scheme that shows the working principle of the evanescent wave-based plasmonic biosensor is also shown. A competitive immunoassay, with a derivative of the analyte is immobilized on the gold sensor surface. The specific antibody together with the free target analyte are found in solution. A competition between the free analyte and the one immobilized on the surface is established for the binding the free antibody, in such a way an inversely proportional signal is obtained (i.e. higher signals are obtained for low analyte concentrations as more antibody is bound to the surface; (B) *(left)* Plot showing the angular scanning which defines the critical angle (θ_{TIR}) which is the minimum angle of incidence at which total internal reflection (TIR) occurs at the interface between the gold and the dielectric (external medium like the liquid samples. Beyond this angle, an evanescent wave is generated, which can excite surface plasmons when resonance conditions are met (SPP or resonance angle θ_{SPP}).a fixed angle (θ) where maximum sensitivity can be achieved is selected to perform the fixed angle measurements in real-time; *(right)* The changes in refractive index (n) as the ones resulting from a biointeraction event on the surface (i.e. antibody-analyte) shifts the resonance angles (from n_1 to n_2) which results in a change in the reflectivity in the real-time analysis. (C) *(left)* Representative sensorgram showing the establishment of interactions in real-time as the free antibody enters the sensing area and interacts with the surface (i.e. antigen), the reach of steady state with stabilized signal, and the addition of a regeneration solution that disrupts the interactions, and leads to the desorption of the antibody and the recovery of the baseline (i.e. antigen-coated surface); *(right)* representative sensorgrams for different samples containing varying concentrations of analyte and a fixed concentration of antibody. Higher analyte concentrations result in lower amount of available antibodies to bind the surface and lower signals.

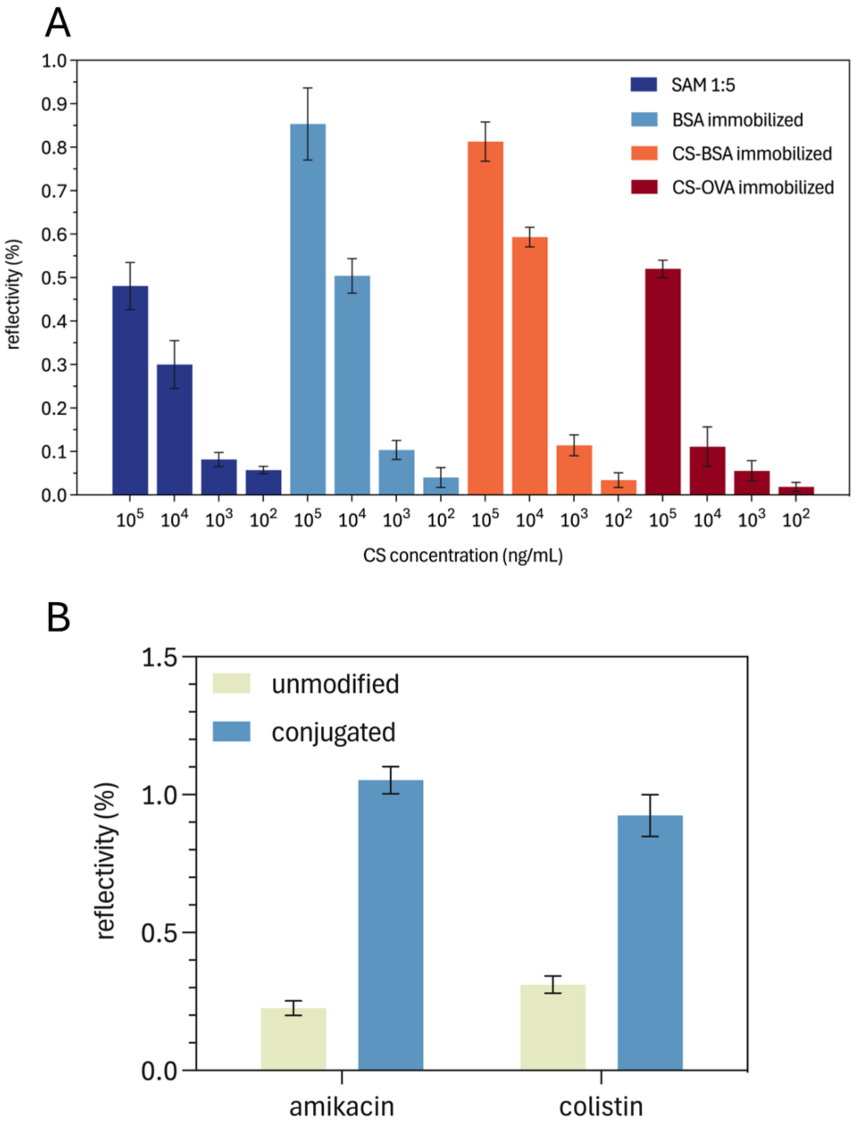

Figure S2. A) Nonspecific adsorptions of colistin over biosensor chips modified: only a self-assembled monolayer (SAM) of thiols; SAM with a layer of unconjugated BSA; SAM with a layer of CS–BSA conjugate and CS–OVA conjugate. Colistin solutions (10^5–10^2 ng/mL) prepared in PBS. No antibody was added; **B)** Antibody response signals for anti–AK and anti–CS at 6 µg/mL over biosensor chips immobilized with amikacin and colistin antibiotics (unmodified, green) or antibiotic conjugates AK–BSA or CS–OVA (blue). Comparison is shown for a total immobilized molecule concentration of 100 µg/mL, as this was found to be optimal for unmodified AK and CS coupling. Data represent the mean ± SD of duplicates

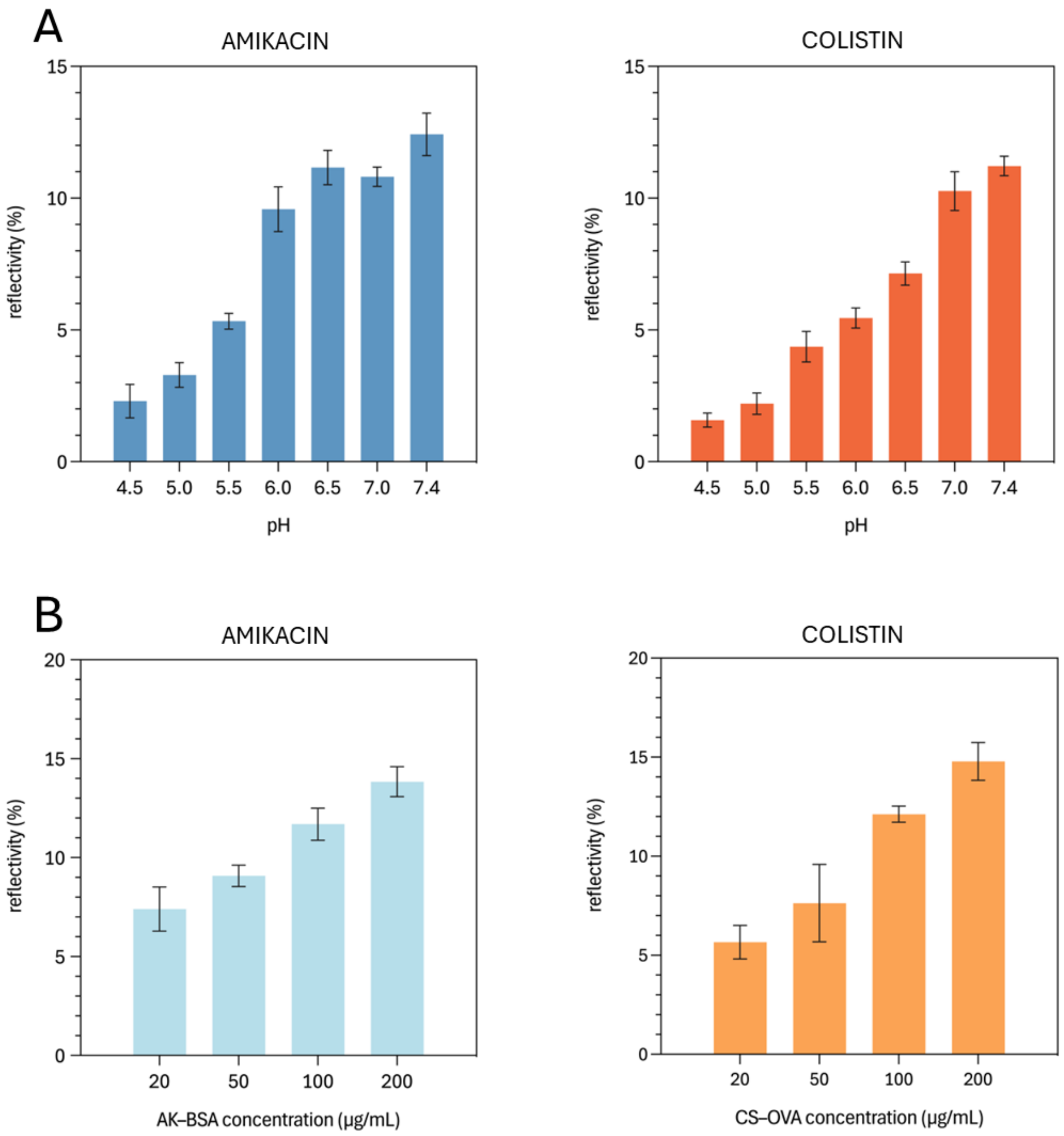

Figure S3. A) Effect of the pH on the immobilization of AK–BSA and CS–OVA conjugates at 100 µg/mL. **B)** Immobilization signals at different concentrations of AK–BSA and CS–OVA. The data correspond to the average ± SD of duplicates.

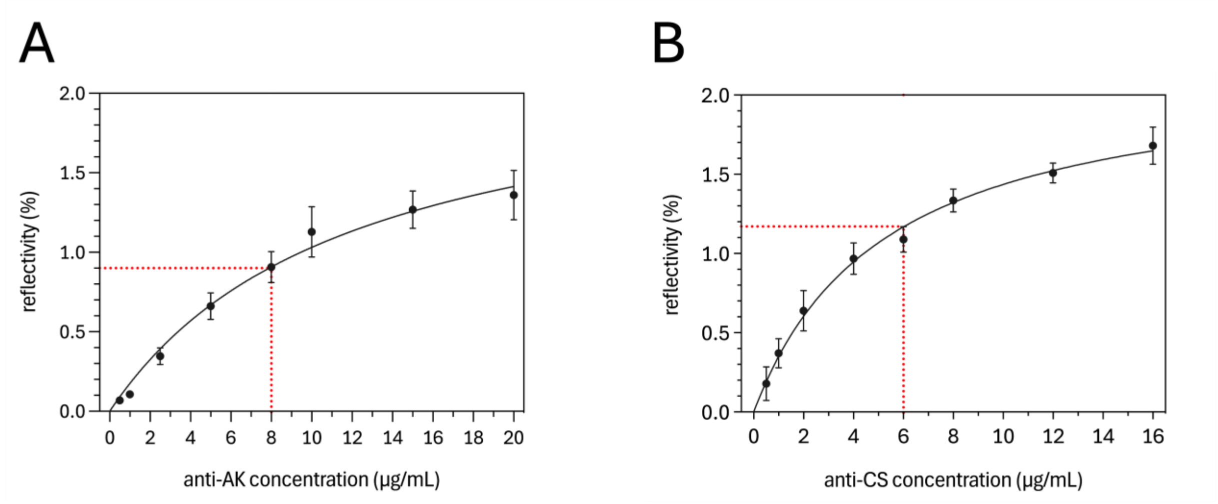

Figure S4. Non-competitive saturation curves for **A)** anti–AK and **B)** anti–CS in PBS with respective conjugates (AK–BSA or CS–OVA) immobilized at 200 μg/mL. Different antibody concentrations are tested in the absence of analyte. The data correspond to the average ± SD of duplicates.

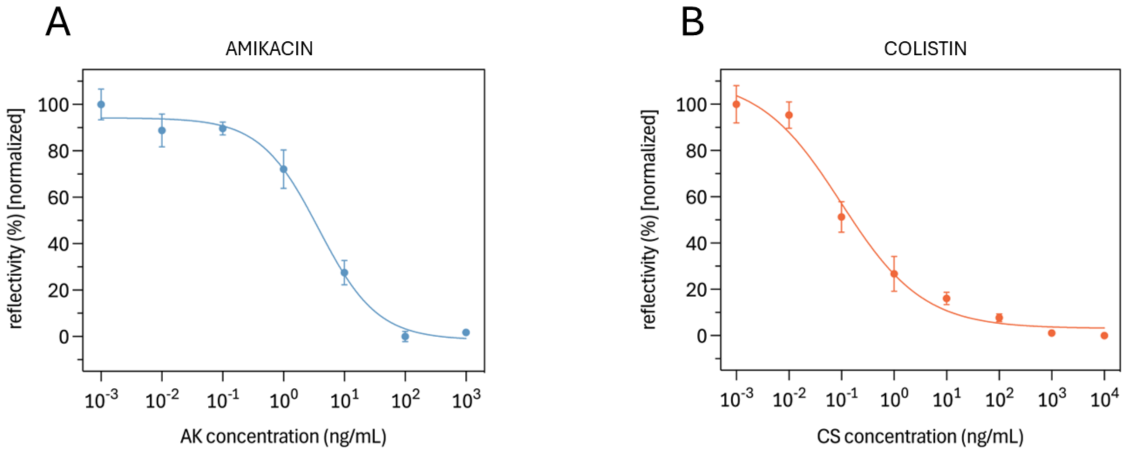

Figure S5. Standard calibration curves in buffer conditions (PBS). **A)** Calibration curve for the amikacin biosensor and **B)** calibration curve for the colistin biosensor. The data correspond to the average ± SD of triplicate measurements.

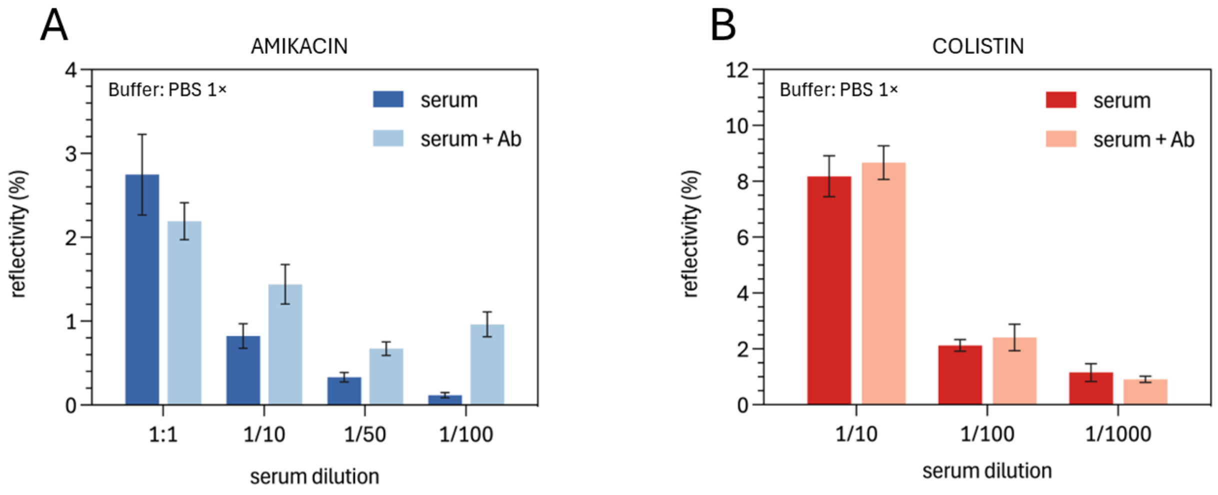

Figure S6. Biosensor response to different serum dilutions without and with each corresponding specific antibody for **A)** amikacin biosensor and **B)** colistin biosensor. [anti–AK] = 8 µg/mL; [anti–CS] = 6 µg/mL. Dilution buffer: PBS. The data correspond to the average ± SD of duplicates.

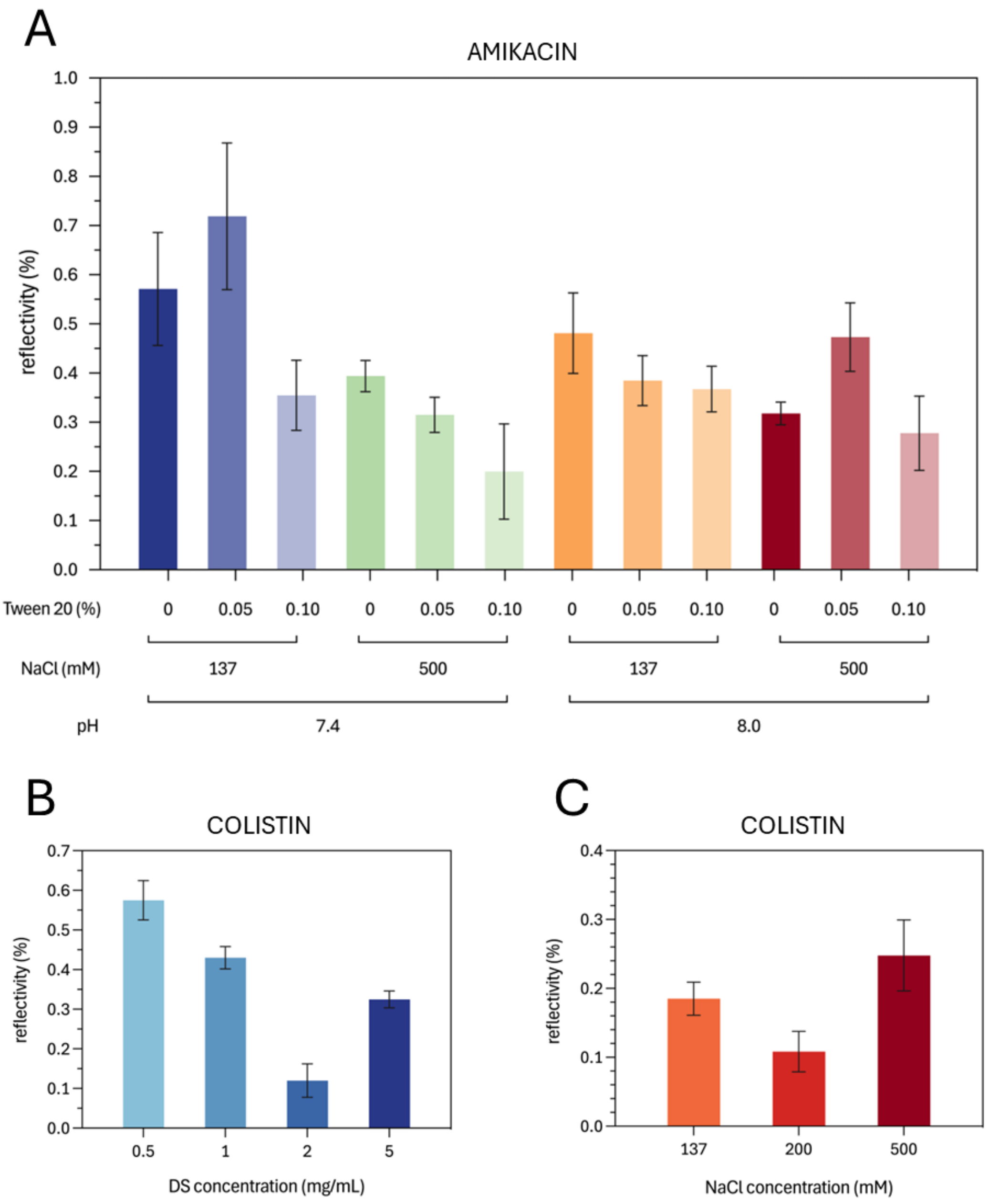

Figure S7. A) Effect of pH and Tween-20 and NaCl concentrations on optimizing the amikacin biosensor immunoassays in 1/10 diluted serum samples to reduce non-specific binding. **B)** Effect of dextran sulfate (DS) concentration on minimizing serum-induced non-specific binding in the colistin biosensor using 1/1000 diluted serum samples. **C)** Effect of NaCl concentration on decreasing non-specific interactions caused by serum in the colistin biosensor, also in 1/1000 diluted serum samples. The combined effect of DS and NaCl can be observed in Figure 6B in the main text. The data correspond to the average ± SD of duplicates.

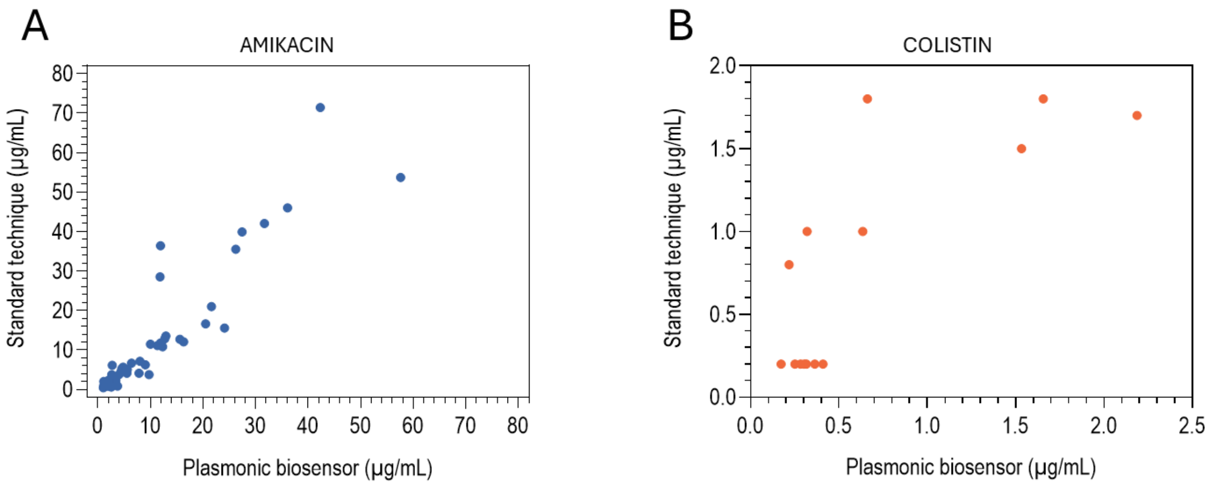

Figure S8. Correlation between the biosensor and measurements with standard techniques for **A)** amikacin biosensor and **B)** colistin biosensor. Spearman's rank correlation coefficients are 0.9171 for the amikacin biosensor and 0.7435 for the colistin biosensor, with respective p-value of < 0.001 and 0.04.

Table S1. Characterization of amikacin and colistin protein conjugates.

**Conjugate molecule**	**AK–BSA**	**CS–BSA**	**CS–OVA**
**Carrier protein weight (Da)**	66,500 (BSA)	66,500 (BSA	44,500 (OVA)
**Analyte weight (Da)**	585.6 (AK)	1174.5 (CS)	1174.5 (CS)
**Conjugate weight (Da)**	73,092	81,725	63,034
**Analyte:protein ratio**	11:1	13:1	16:1

Table S2. Biosensor-based competitive immunoassay parameters for amikacin and colistin in buffer*.

	**Amikacin biosensor**¶	**Colistin biosensor**¶
**LOD (IC_{90})**	0.12 ± 0.07 ng/mL<br>(0.20 ± 0.12 nM)	8.04 ± 6.24 pg/mL<br>(6.85 ± 5.31 pM)
**IC_{50}**	3.20 ± 1.65 ng/mL<br>(5.46 ± 2.81 nM)	160.47 ± 23.55 pg/mL<br>(136.62 ± 20.05 pM)
**Working range (IC_{80} – IC_{20})**	0.55 ± 0.27 – 14.96 ± 7.60 ng/mL<br>(0.94 ± 0.46 – 25.55 ± 12.97 nM)	19.87 ± 9.34 – 2045.63 ± 208.94 pg/mL<br>(16.92 ± 7.95 – 1741.65 ± 177.89 pM)
**Slope**	-0.90 ± 0.19	-0.56 ± 0.15

*PBS pH 7.4
Data obtained from triplicate measurements

Table S3. Blocking agents study for the amikacin biosensor to minimize non-specific binding in serum 1/10.

**Blocking agent**	**Concentration**	**ΔR (%)**	
		**Serum response***	**anti-AK response†**
No blocking agents	-	0.82 ± 0.15	0.62 ± 0.19
PLL-PEG	0.1 mg/mL	0.65 ± 0.04	0.52 ± 0.02
	0.2 mg/mL	0.51 ± 0.05	0.55 ± 0.05
	0.5 mg/mL	0.31 ± 0.02	0.64 ± 0.23
BSA	1%	0.40 ± 0.02	0.69 ± 0.16
	2%	0.26 ± 0.01	0.43 ± 0.01
	5%	0.08 ± 0.01	0.36 ± 0.03
OVA	2%	0.14 ± 0.02	0.49 ± 0.03

*Signals obtained in 1/10 serum dilution in PBS
†Signals obtained in PBS
Samples were measured by duplicate

Table S4. Blocking agents study for the colistin biosensor to minimize non-specific binding in serum 1/1000.

Blocking agent	Concentration	ΔR (%)	
		**Serum response***	**anti-CS response†**
No blocking agents	-	1.15 ± 0.23	N/A‡
PLL-PEG	0.2 mg/mL	0.57 ± 0.05	0.15 ± 0.04
	0.5 mg/mL	0.47 ± 0.12	0.12 ± 0.10
BSA	1%	1.35 ± 0.23	0.19 ± 0.08
	2%	1.52 ± 0.11	0.11 ± 0.05
OVA	1%	0.86 ± 0.04	0.28 ± 0.12
	2%	1.70 ± 0.33	0.10 ± 0.06

*Signals obtained in 1/1000 serum dilution in PBS

†Signals obtained in PBS

‡Antibody signal masked

Samples were measured by duplicate

Table S5. Intra-assay and inter-assay variability of the key analytical parameters for the plasmonic-based assays conducted in diluted serum

	Amikacin biosensor†			
	Intra-assay*		Inter-assay**	
**Parameter**	**Mean ± SD**	**% CV**	**Mean ± SD**	**% CV**
**LOD (ng/mL)**	0.75 ± 0.09	12.1	0.94 ± 0.16	17.0
**IC_{50} (ng/mL)**	11.9 ± 2.52	21.1	11.1 ± 2.18	19.6
**ΔR_{max} (%)**	1.50 ± 0.02	1.34	1.55 ± 0.08	5.16
**Hill slope**	-0.97 ± 0.17	17.5	-1.04 ± 0.19	18.3
	**Colistin biosensor‡**			
	**Intra-assay**		**Inter-assay**	
**Parameter**	**Mean ± SD**	**% CV**	**Mean ± SD**	**% CV**
**LOD (pg/mL)**	3.49 ± 0.31	8.70	3.95 ± 0.63	15.9
**IC_{50} (pg/mL)**	27.40 ± 5.12	18.7	36.79 ± 8.93	24.3
**ΔR_{max} (%)**	0.81 ± 0.05	6.95	0.82 ± 0.01	1.22
**Hill slope**	-0.76 ± 0.08	10.5	-0.69 ± 0.09	13.0

†1/10 serum dilution in PBST 0.10% pH 7.4 500 mM NaCl

‡1/1000 serum dilution in PBS pH 7.4 200 mM NaCl + DS 2 mg/mL

* Samples were measured by duplicate in the same biosensor chip.

** Samples were measured by triplicate, two from the same biosensor chip and one from a different biosensor chip.

Table S6. Quantification of amikacin concentrations in human serum samples.

Sample	Reported concentration (μg/mL)	Plasmonic biosensor (μg/mL)*	Accuracy (%)
04 8515309 2	N/A	(15.3 ± 1.4)	N/A
14 6519223 9	MHEM	(5.9 ± 3.6)	N/A
13 8505138 8	28.5	(11.8 ± 2.3)	41
04 7269296 9	6.1	(2.7 ± 0.4)	44
04 7901435 3	2.0	(1.1 ± 0.3)	55
13 7265815 7	71.4	(42.3 ± 11.3)	59
14 6855617 7	1.8	(1.2 ± 0.5)	67
13 8501457 1	39.9	(27.4 ± 3.1)	69
14 8501455 7	3.7	(2.6 ± 1.2)	71
13 7897588 0	35.5	(26.2 ± 8.4)	74
13 7807263 4	42.0	(31.7 ± 7.3)	75
13 8520758 2	46.0	(36.1 ± 6.7)	78
04 5220708 8	16.6	(20.5 ± 4.1)	81
04 6292405 0	3.3	(2.7 ± 1.4)	82
04 7269084 1	5.6	(4.8 ± 4.1)	86
04 7683729 4	5.2	(4.5 ± 0.6)	87
04 8181907 0	2.3	(2.0 ± 0.7)	87
14 6853244 8	11.4	(10.0 ± 2.8)	88
04 8507243 2	5.1	(4.5 ± 0.2)	90
13 7269079 4	53.7	(57.6 ± 21.2)	93
04 7897868 5	13.5	(12.9 ± 5.8)	96
04 7805092 5	6.6	(6.4 ± 1.5)	97
04 5221707 5	12.8	(12.7 ± 8.1)	99
04 7818036 5	4.5	(4.4 ± 2.4)	98
14 7688226 4	36.4	(11.9 ± 2.0)	98
04 8513875 1	11.1	(11.3 ± 2.4)	102
13 7265271 0	21	(21.6 ± 6.4)	103
14 7688226 4	11.7	(11.9 ± 2.0)	102
14 8242677 3	1.4	(1.5 ± 0.5)	107
14 7265817 5	3.7	(4.0 ± 1.4)	108
14 6852727 4	1.0	(1.1 ± 0.4)	110
04 7265085 8	1.6	(1.8 ± 0.4)	113
14 5977462 1	5.0	(5.6 ± 2.8)	112
04 7808467 6	10.8	(12.3 ± 3.0)	114
04 7267695 0	7.1	(8.0 ± 2.9)	113
14 7902721 9	12.7	(15.6 ± 3.9)	123
14 7265272 0	2.0	(2.5 ± 1.0)	125
14 8521947 7	< 0.8	(1.0 ± 0.2)	125
14 7269078 8	< 0.8	(1.0 ± 0.1)	125
14 6286907 6	4.2	(5.6 ± 1.3)	133
14 7271651 5	0.9	(1.2 ± 0.5)	133
14 7271651 5	0.9	(1.2 ± 0.5)	133
14 7821167 9	< 0.8	(1.1 ± 0.1)	138
04 7815902 1	1.4	(1.9 ± 0.2)	136
04 8512751 2	<0.8	(1.1 ± 0.1)	138
14 8196175 6	4.1	(5.5 ± 0.7)	134
04 6848407 8	12	(16.3 ± 5.1)	136
04 7266969 9	15.5	(22.1 ± 1.5)	142
14 7265085 9	1.6	(2.3 ± 0.9)	144
14 6910216 1	6.2	(9.0 ± 2.1)	145

Sample	Reported concentration (µg/mL)	Plasmonic biosensor (µg/mL)*	Accuracy (%)
14 7271860 7	< 0.8	(1.2 ± 0.8)	150
14 7898992 0	2.2	(3.4 ± 1.7)	155
14 7807262 8	< 0.8	(1.5 ± 0.8)	188
14 7265095 6	4.1	(7.8 ± 2.2)	190
13 8507862 6	< 0.8	(1.8 ± 0.2)	225
14 7271651 5	0.9	(2.1 ± 0.7)	233
14 7428390 0	< 0.8	(1.9 ± 0.3)	238
14 7817966 0	< 0.4	(1.0 ± 0.1)	250
14 8501455 7	3.7	(9.7 ± 1.6)	262
14 8073846 3	0.9	(2.5 ± 1.2)	278
14 6911637 5	1.1	(3.2 ± 1.7)	291
14 7682009 0	< 0.8	(2.5 ± 1.4)	313
04 7831634 8	0.6	(2.5 ± 0.3)	417
14 7682009 0	< 0.8	(3.7 ± 0.1)	463

* Samples were measured by duplicate.

Table S7. Quantification of colistin concentrations in human serum samples.

Sample	Reported concentration (μg/mL)	Plasmonic biosensor (μg/mL)*	Accuracy (%)
73 5210050 4	0.8	(0.22 ± 0.12)	27
51 5211266 3	1	(0.32 ± 0.22)	32
51 5211803 3	1.8	(0.67 ± 0.23)	37
73 5210697 9	1	(0.63 ± 0.20)	63
73 7815906 9	< 0.2	(0.17 ± 0.02)	87
73 5211804 7	1.8	(1.65 ± 0.61)	92
72 7576671 0	1.5	(1.53 ± 0.86)	102
73 7742179 7	< 0.2	(0.25 ± 0.07)	125
51 5212645 1	1.7	(2.19 ± 0.54)	129
73 7739200 2	< 0.2	(0.28 ± 0.05)	141
73 7806441 6	< 0.2	(0.30 ± 0.18)	152
72 8494985 6	< 0.2	(0.31 ± 0.08)	158
73 8511736 8	< 0.2	(0.36 ± 0.08)	181
73 7696245 0	< 0.2	(0.41 ± 0.09)	205

* Samples were measured by duplicate.

Table S8. Bland-Altman analysis and performance metrics for the amikacin and colistin biosensors.

	**AK biosensor**	**CS biosensor**
**Mean difference (µg/mL)**	0.1467	64.4091
**SD of differences (µg/mL)**	3.4203	202.7051
**95% limits of agreement (µg/mL)**	[-6.7944 – 6.5010]	[-461.7111 – 332.8929]
**t-statistic**	-0.3322	-1.0048
**p-value**	0.7409	0.3387
